# Defining patients living long-term with incurable cancer: A modified hybrid Delphi study

**DOI:** 10.1177/02692163251400114

**Published:** 2025-12-26

**Authors:** Ruben Bouma, Mariken E. Stegmann, Natasja J. H. Raijmakers, Lia van Zuylen, Anna K. L. Reyners, Kristel van Asselt, Maatje D. A. van Gastel, Daan Brandenbarg, Olaf P. Geerse

**Affiliations:** 1 University Medical Center Groningen, Department of Primary and Long-Term Care, University of Groningen, The Netherlands; 2 Department of Research & Development, Netherlands Comprehensive Cancer Organization (IKNL), Utrecht, The Netherlands; 3 Medical Oncology, Amsterdam UMC location Vrije Universiteit Amsterdam, The Netherlands; 4 Treatment and Quality of Life, Cancer Center Amsterdam, The Netherlands; 5 Department of Medical Oncology, University Medical Centre Groningen, University of Groningen, The Netherlands; 6 Department of General Practice and Nursing Science, Julius Center for Health Sciences and Primary Care, University Medical Center Utrecht, The Netherlands; 7 Department of Hematology, University Medical Center Groningen, University of Groningen, The Netherlands; 8 Department of Pulmonary Diseases, Amsterdam UMC location Vrije Universiteit Amsterdam, The Netherlands

**Keywords:** cancer, oncology, incurable, long-term, definition, modified hybrid Delphi

## Abstract

**Background:**

Therapeutic advances have significantly extended survival for certain groups of patients with incurable cancer, creating a growing population living long-term with incurable cancer. However, the absence of standardized definitions and terminology has contributed to limited recognition of this distinct group and their specific care needs.

**Aim:**

To achieve consensus on definitions and terminology for patients living long-term with incurable cancer by incorporating perspectives of patients, informal caregivers, healthcare professionals, and other relevant stakeholders.

**Design:**

A modified hybrid Delphi study, comprising focus groups and a three-round Delphi consensus process.

**Setting/participants:**

Three focus groups were conducted with patients (*n* = 11), informal caregivers (*n* = 4), and healthcare professionals (*n* = 6). The multidisciplinary expert group comprised medical specialists (*n* = 5), epidemiologists (*n* = 3), and patient advocates (*n* = 2). The Delphi study involved 78 panelists (73 unique respondents) divided into three subpanels: patients and informal caregivers (*n* = 22), healthcare professionals (*n* = 36), and other stakeholders (*n* = 20). All participants were from the Netherlands.

**Results:**

We achieved 88% consensus on the terminology: Patients living long-term with incurable cancer. Consensus was reached on the definition (94%) for patients living for two or more years with: (1) incurable metastatic cancer, (2) incurable hematological malignancies, (3) incurable locally advanced cancer, or (4) patients with exceptionally long survival for their cancer type, despite not meeting the 2-year criterion.

**Conclusions:**

This modified hybrid Delphi study established the first consensus-based framework for patients living long-term with incurable cancer, providing essential groundwork for improved recognition and tailored care approaches for this population.

**What is already known about the topic?**

- Therapeutic advances in cancer treatment have increased survival for certain incurable cancer types, leading to a growing population living long-term with incurable cancer.
- The absence of standardized definitions and terminology hinders identification and characterization of this group, thereby limiting tailored care and policy development.

**What this paper adds?**

- Provides the first systematically developed consensus-based framework to define and establish terminology for patients living long-term with incurable cancer.
- Demonstrates a robust methodological approach (modified hybrid Delphi) that enhances the validity and applicability of the definition.
- Highlights the importance of integrating diverse stakeholder perspectives, including patients, caregivers, and healthcare professionals, in defining this patient group.

**Implications for practice, theory, or policy**

- This framework provides a foundation for future epidemiological research.
- Standardized terminology can enhance recognition and improves communication between patients, caregivers, and healthcare professionals regarding this distinct group.
- By clearly defining this patient population, our framework could potentially support the development of clinical guidelines and policies tailored to patients living long-term with incurable cancer.

## Introduction

Cancer remains a major global health challenge, with nearly 20 million new cases and 10 million deaths reported worldwide in 2022.^1^ In the European Union, cancer incidence is projected to rise from 2.74 million in 2022 to 3.25 million in 2040, with cancer-related deaths increasing by over 24% by 2035.^2,3^

Palliative care is advocated for all patients with cancer, but certainly for those with an incurable disease.^4,5^ However, this incurable group becomes more heterogeneous. While metastatic cancer has traditionally been considered incurable, with prognosis varying by subtype,^6^ recent therapeutic advances, particularly in immunotherapy and targeted therapies including EGFR inhibitors, have extended survival in certain cancers.^6–9^ Notable examples include metastatic melanoma with 5-year survival rates nearing 30%, and metastatic breast and prostate cancers with 3-year survival rates approaching 50%.^10,11^ However, progress remains limited for other cancer types.^8,11^ Additionally, the emerging concept of oligometastatic disease challenges the binary classification of metastatic cancer as incurable versus curable, as curative treatment may be feasible in select cases depending on the number, site, and/or location of metastasis.^12^ As a result, a growing population of patients survives for extended periods of time with a cancer that is likely incurable, necessitating a clearer understanding of their care needs. Definitions of survivorship and palliative care trajectories vary across countries and health systems, highlighting the need for internationally relevant frameworks to identify and support this growing group.^13^ Patients living long-term with incurable cancer form a key population for whom palliative care should be a standard part of care, yet this is not consistently embedded in practice, partly due to the limited knowledge and recognition of this growing group.^4^

Patients living long-term with incurable cancer face complex challenges across emotional, psychosocial, physical, and spiritual domains.^14–19^ Patient advocates have increasingly emphasized the need for more comprehensive efforts to address these growing and unmet supportive care needs.^20–22^ Furthermore, many patients, as well as their informal caregivers, report feeling caught between two worlds: neither receiving care tailored to end-of-life needs, nor fitting the typical narrative of cancer survivorship. This in-between state has been described as a “twilight zone,” where patients are aware that treatment is life-prolonging but not curative, and that death is near. Yet, they may live for years, navigating ongoing shifts between illness, stability, and uncertainty.^16,18^ Consequently, they receive insufficiently tailored support from various healthcare providers, including oncologists, general practitioners, and allied healthcare professionals.^5,14,23^ Yet, the lack of a clear consensus-based definition and standardized terminology for this patient population continues to drive persistent gaps in care.^4,23^

Existing studies use varying definitions to describe patients living long-term with incurable cancer, ranging from ⩾2 years progression-free survival or ⩾3 years overall survival to stable disease or median time since treatment initiation in qualitative studies.^15,16,24^ Terminology also varies widely, as shown in the scoping review by Kolsteren et al., with terms such as “advanced metastatic cancer,” “long-term responder,” and “metavivorship” used inconsistently.

To address this critical issue, we conducted a modified hybrid Delphi study comprising focus groups, expert consultations, and a three-round Delphi consensus process to develop a standardized framework consisting of a definition and terminology for patients living long-term with incurable cancer.

## Methods

### Study design

We conducted a modified hybrid Delphi study to establish consensus on both the definition and terminology for patients living long-term with incurable cancer. This approach combined focus groups, expert consultations, and a three-round Delphi process.^25,26^ Unlike the traditional hybrid Delphi approach,^27^ we replaced the Nominal Group Technique with iterative expert consultations to enable refinement after the focus group analysis and following Delphi Round 2. The definition refers to the criteria for identifying this patient group, while terminology addresses language used in clinical and layman’s contexts. Together, these consensus-based elements form a framework. The Consolidated Criteria for Reporting Qualitative Research guidelines were followed to report the methods and findings of the focus groups.^28^ The Medical Ethics Review Committee exempted the study from the Dutch Medical Research Involving Human Subjects Act (WMO), and formal ethical approval was not required (2023/575).

### Research objective

**Figure 1.**
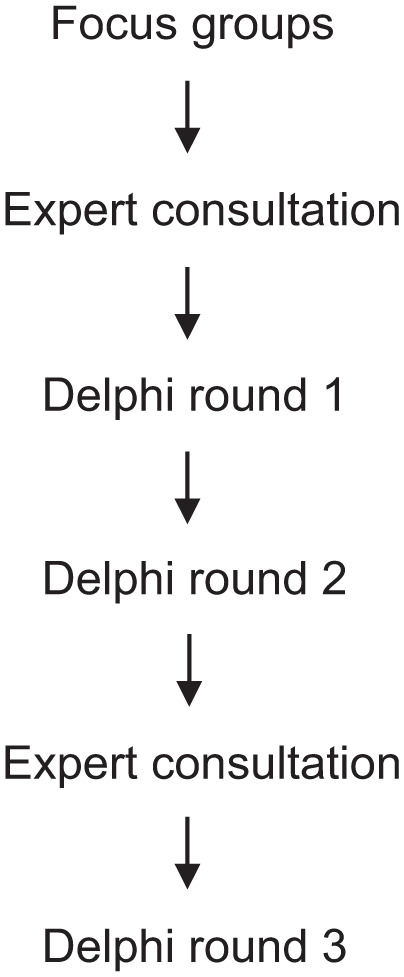
Study timeline.

The aim was to reach consensus on standardized definitions and terminology for this patient population. Focus groups with patients, informal caregivers, and healthcare professionals explored perspectives on “long-term,” “incurable,” and terminology. Findings informed expert consultations that shaped the Delphi design and guided the formulation of a preliminary definition and terminology for final evaluation in Round 3. An overview of the study timeline is provided in Figure 1.

### Focus groups

#### Population and sampling

The study included adults (⩾18 years) with incurable cancer, their informal caregivers, and (allied) healthcare professionals involved in their care. All participants were required to be able to understand and speak Dutch. Patients had received their incurable cancer diagnosis at least 6 months prior, while informal caregivers had experience supporting someone with such a diagnosis. All were asked to reflect beyond their personal experiences. Purposive sampling ensured variation in gender, age, cancer type, and time since diagnosis (patients), and discipline and experience (healthcare professionals). Sampling and recruitment were iterative to ensure diversity.

#### Recruitment

Patients and caregivers were recruited via flyers at Institutions for Psychosocial Oncology – Center for Life with and after cancer, via the Dutch patient website kanker.nl, and social media. Healthcare professionals were approached through professional networks, direct invitations, and LinkedIn. Informed consent was obtained prior to participation, either in written form for in-person focus groups or digitally for online sessions.

#### Data collection

Using literature, a semi-structured topic guide was developed on terminology, definitions of “long-term” and “incurable,” and relevant subgroups. Input from a medical oncologist (LZ) informed fictional case vignettes. Four main themes guided discussions: (1) definition of “long-term” and timing, (2) definition of “incurable,” (3) terminology, and (4) relevant subgroups. Interactive methods (e.g., post it notes, sticker voting) encouraged engagement. The topic guide is available in Supplemental File 1. Each focus group session lasted between 90 and 120 min. One male researcher (RB) with limited experience moderated discussions, supported by experienced qualitative researchers who are also physicians (MS and/or OG), who also took field notes. Sociodemographic and professional data were collected via REDCap.^29^ Patients reported cancer type and time since diagnosis, informal caregivers reported their relationship, and healthcare professionals reported discipline and experience.

#### Data analysis

Discussions were recorded, transcribed verbatim, and analyzed thematically using a hybrid inductive-deductive approach.^30^ Codes were derived from the topic guide and refined through analysis. Three researchers (RB, MS, OG) independently coded transcripts in ATLAS.ti 24.0,^31^ resolving discrepancies through consensus. A code tree was developed iteratively, balancing research aims with emerging themes.

### Expert consultations

A multidisciplinary group consisting of medical specialists (LZ, AR, OG), general practitioners (MS, KvA), epidemiologists (NR, DB, MS), and two patient advocates (with hematology expertise) met twice. Based on the focus group findings, they refined thematic classifications and designed the Delphi study. They decided to submit only terminology and definitions for consensus, excluding subgroup classifications. After Round 2 of the Delphi study, they reviewed interim results and formulated a preliminary definition and terminology for final evaluation.

### Delphi study

#### Composition of the subpanels

Three subpanels were formed: (1) patients/informal caregivers, (2) healthcare professionals, and (3) other stakeholders (e.g. researchers, oncology/palliative care advisors, organizational leaders). Some participants were included in multiple panels, for example, healthcare professionals who were also informal caregivers. Their responses were included in both relevant subpanel terminology analyses in Round 1. Patients and informal caregivers were recruited from individuals who had previously expressed interest in focus groups. Healthcare professionals, including oncologists, general practitioners specialized in palliative care, and allied professionals, were selected based on their involvement in care for this patient population. Other stakeholders were affiliated with relevant organizations.

#### Study conduct and data collection

The Delphi study was conducted via REDCap,^29^ following established guidelines.^25^ Participants received study information, consented digitally, and completed online questionnaires in Dutch. Non-responders were reminded twice. Limited sociodemographic data were collected (e.g., age, sex, education, profession).

#### Delphi Round 1

Two questionnaires were developed; one for professionals/stakeholders (medical terminology) and one for patients/informal caregivers (layman terminology), with simplified language where appropriate. Round 1 included 22 items on defining “incurable” and “long-term” and appropriate terminology, rated on a 5-point Likert scale with space for comments. All relevant themes and perspectives raised during the focus groups, were translated into Delphi items to ensure systematic follow-up and enable iterative consensus building. Items with median scores ⩾ 4 reached consensus; <2 were excluded; those between 2 and 4 were deemed relevant, revised, and resubmitted (see Supplemental File 2).

#### Delphi Rounds 2 and 3

Items deemed relevant in Round 1 were reformulated and reassessed in Round 2, where all panelists received the same questionnaire and indicated agreement (yes/no). Consensus was defined a priori as ⩾70% agreement across all panels.^25^ Based on results, the expert group drafted a preliminary definition and terminology, which were re-evaluated in Round 3 using the same threshold. The final terminology was translated into English by a native speaker after the consensus process.

#### Analysis

Data were exported from REDCap to Microsoft Excel (version 25). Open-ended suggestions mentioned by ⩾3 participants were included in subsequent rounds, while terms perceived as emotionally discomforting were excluded. Descriptive statistics summarized the data.

## Results

### Focus groups

Three focus groups were held between March and May 2024; two in-person sessions with a total of 11 patients with solid tumors and four informal caregivers, and one online session with six healthcare professionals. Participant characteristics for the focus groups are presented in Table 1.

**Table 1.** Characteristics of focus group participants (*n* = 21).

	Patients with incurable cancer (*n* = 11)	Informal caregivers (*n* = 4)	Healthcare professionals (*n* = 6)
Characteristics	*N* (%)	*N* (%)	*N* (%)
Sex			
Female	6 (55)	1 (25)	4 (66)
Age (years)			
18–50	1 (9)	1 (25)	3 (50)
51–75	8 (73)	3 (75)	3 (50)
75+	2 (18)	0	0
Formal education^a^			
Less	0	0	0
Intermediate	6 (55)	1 (25)	0
More	5 (45)	3 (75)	6 (100)
Ethnicity			
Dutch	10 (91)	3 (75)	6 (100)
Other	1 (9)	1 (25)	0
Type of cancer			
Prostate	3 (27)		
Colorectal	2 (18)		
Breast	2 (18)		
Other	4 (36)		
Time since diagnosis (years)			
1–2	5 (45)		
2–5	3 (27)		
>5	3 (27)		
Relation to patient			
Partner		3 (75)	
Sibling		1 (25)	
Discipline			
Medical oncologist			2 (33)
Pulmonologist-oncologist			1 (17)
Radiation therapist			1 (17)
Physician assistant			1 (17)
General practitioner			1 (17)
Work experience (years)			
5–10			2 (33)
10–20			1 (17)
>20			3 (50)

a Education levels categorized according to the ISCED classification: less formal education = ISCED 0–2 (primary to lower secondary), intermediate formal education = ISCED 3–4 (upper secondary to post-secondary non-tertiary), and more formal education = ISCED 5–8 (tertiary education: bachelor’s, master’s, doctorate).^30^

#### Definition

Participants generally considered cancer with multiple metastases to be incurable, except in types where metastases are known to be potentially curatively treatable, such as testicular cancer. The inclusion of patients with oligometastatic disease was debated; many felt they should be excluded when curative treatment is possible, which is based on the number and location of metastases, as well as the patient’s overall fitness (to receive the curative treatment; Quote 1 (Q1)).

Most participants supported including patients with locally advanced, non-metastatic tumors that are not amenable to curative treatment, given their poor prognosis and the non-curative intent of treatment. Opinions diverged regarding patients without metastases who either refuse curative treatment for personal reasons or cannot receive it due to comorbidities. Some felt these individuals should be excluded, as curative treatment was technically available but not pursued, while others argued they should be included, as the refusal or ineligibility renders their disease effectively incurable. Hematological malignancies without curable options were considered to fit the definition, though participants highlighted the need for further clarification due to the heterogeneous and sometimes indolent course of these conditions.

Some participants linked prolonged survival to exceeding the palliative phase, suggesting a minimum life expectancy of over 1 year (Q2). Some proposed a survival threshold of 2–5 years, aligning with monitoring practices in cancers like melanoma (Q3). A relative definition was also suggested, adjusting for cancer type, since fixed survival thresholds may exclude patients with aggressive types of cancers who may still live for a relatively long time despite their poor prognosis.

In lung cancer, I would consider this [oligometastatic disease with liver metastasis] as stage 4 [incurable], unless I have a very healthy, young patient in front of me. (Q1. Healthcare professional)

We use the surprise question: Would I be surprised if this patient were alive in a year? (. . .) If the answer is yes, they are not palliative and could belong to this group. (Q2. Healthcare professional )

The first five years are monitored intensively. (. . .) If the response is sustained over time, I think a duration of more than two years is certainly needed, and I believe that five years is a reasonable timeframe (Q3. Healthcare professional)

#### Terminology

Patients explored the preference for a mix of layman and medical terminology. Terms like *Tussenlanders* (freely translated to “people living in the place in between”) were viewed as empowering. However, participants warned against using language that might carry stigmatizing implications (Q4). While some suggested the use of distinct terms for laymen and medical communication, others emphasized the importance of a single term that is both clear and self-explanatory. Terms like “prolonged” and “long-term” were considered essential to emphasize both the chronic but life-limiting aspects of living with this disease (Q5). As “metastatic” is not applicable to hematology, participants considered a more inclusive term (Q6).

When I use ‘palliative,’ patients associate it with ‘I might die tomorrow,’ compared to ‘incurable,’ which carries less weight. (Q4. Healthcare professional)

The word ‘prolonged’ is particularly important. (. . .) An initial prognosis of metastasis is often rather hopeless, making you feel like you might not be here in a year. Yet, in the end, you are still here. And with significant advances in science, cancer can sometimes remain incurable but prolonged. (Q5. Patient – FG1)

The term ‘metastatic’ is strictly for oncology. (. . .) If this definition includes hematology, it would not be appropriate. (Q6. Healthcare professional)

#### Subgroups

Most participants acknowledged the potential usefulness of defining subgroups (e.g., patients receiving treatment vs watchful waiting) but preferred a flexible approach that prioritizes tailored care, as patients’ needs will likely vary over time (Q7).

I actually think a selection menu is a nice idea. Or you could have certain reference points at set intervals, for example, every few months, where the patient can indicate: ‘I would now like support with this or that,' or 'I'm doing better, so this can be discontinued.’ (Q7. Patient – FG1)

### Delphi study

#### Composition of the subpanels

A total of 78 panelists (73 unique respondents) participated in the Delphi study across three subpanels: patients and informal caregivers (*n* = 22), healthcare professionals (*n* = 36), and other stakeholders (*n* = 20), including researchers, advisors, directors, and a patient advocate. Five individuals participated in more than one subpanel in Round 1 due to overlapping roles, as detailed in the Methods section. All panelists completed Round 1, 90% participated in Round 2, and 88% in Round 3. Detailed panel characteristics are presented in Table 2.

**Table 2.** Characteristics of unique Delphi panelists (*n* = 73).

	Patients and informal caregivers (*n* = 17)	Healthcare professionals (*n* = 36)	Relevant stakeholders (*n* = 20)
Characteristics	*N* (%)	*N* (%)	*N* (%)
Sex			
Female	10 (59)	20 (56)	18 (90)
Age (years)			
18–50	3 (18)	17 (47)	12 (60)
51–75	14 (82)	19 (53)	8 (40)
75	0	0	0
Situation			
Patient	13 (76)		
Informal caregiver^a^	4 (24)		
Discipline			
Pulmonologist		5 (14)	
Medical oncologist		5 (14)	
Nurse specialist /physician assistant		5 (14)	
Surgeon		4 (11)	
Other		17 (47)	
Stakeholder role			
Researcher			11 (55)
Management^b^			4 (20)
Senior oncology/palliative care advisor			4 (20)
Patient advocate			1 (5)

a Characteristics of healthcare professionals (*n* = 2) and relevant stakeholders (*n* = 3) who were also informal caregivers are presented only within their respective subpanel characteristics.

b Management includes directors or chairs of organizations involved in oncology or palliative care.

#### Definition

In Round 1 (September–November 2024), panelists assessed 16 definition-related items, achieving consensus on two items while 14 items were deemed relevant (Supplemental File 2). Based on comments, these were refined (by RB, MS, DB, OG) into six revised items for Round 2 with one additional item introduced. Round 2 (December 2024) achieved consensus on four items, notably excluding patients with oligometastatic disease and a favorable prognosis. The items and results of Round 2 are displayed in Table 3.

**Table 3.** Items and results Delphi Round 2.

Round 2	Total panel (*n* = 66; %)	Patients & informal caregivers (*n* = 16; %)	Healthcare professionals (*n* = 34; %)	Relevant stakeholders (*n* = 16; %)
Definition				
*Do you agree with the inclusion of the following groups within the definition?*				
Patients who are still alive 2 years after being diagnosed with incurable cancer.	75	81	72	71
Patients who are still alive 5 years after being diagnosed with incurable cancer.	87	100	83	87
No fixed time limit should be set for inclusion.	91	94	88	84
Patients without metastatic disease who are unable to undergo curative treatment (e.g., due to comorbidities).	85	75	97	64
Patients without metastatic disease who choose not to undergo curative treatment (e.g., due to unacceptable side effects affecting their quality of life).	65	64	71	50
Patients who survive exceptionally long relative to the typical prognosis for their cancer type.	81	88	78	81
*Do you agree with the exclusion of the following group from the definition?*				
Patients with oligometastatic disease and a favorable prognosis who are eligible for potentially curative treatment (with up to five metastases in addition to the primary tumor).	76	71	71	92
Terminology				
Both *patients* and *persons* can be used. Which term do you prefer? Percentage for *patient*s is displayed.	61	38	80	38
*Do you agree with the use of one or more of the following terms?*				
*Multiple options may be considered appropriate.*				
Patients with long-term incurable cancer	64	75	56	69
Patients with long-term advanced cancer	18	13	18	25
Long-term survivors with incurable cancer	38	31	38	44
Patients with chronic cancer	27	44	26	13
Patients with a chronic form of advanced cancer	21	19	24	19
Patients with long-term advanced cancer and an uncertain prognosis	18	0	21	31
Patients with long-term incurable cancer and an uncertain prognosis	36	38	35	38
Patients with incurable cancer and a chronic, uncertain prognosis	18	19	18	19
Patients experiencing long-term uncertainty due to advanced cancer	14	0	19	38
Patients experiencing long-term uncertainty due to incurable cancer	23	25	0	44

A draft definition was presented in Round 3 (January 2024), after which 94% of the total panel (range: 88%–97% across subpanels) agreed with the proposed definition. Minor linguistic refinements were made based on feedback. The final definition is presented in Box 1. It includes patients who have lived for 2 or more years with (1) incurable metastatic cancer, (2) incurable hematological malignancies, (3) locally advanced cancer (e.g., tumors invading major blood vessels), and (4) those living exceptionally long for their specific incurable cancer type, despite not meeting the 2-year criterion.

**Box 1.** Final proposed framework for patients/persons living long-term with incurable cancer.

Terminology:
Patients/persons living long-term with incurable cancer
Definition:
The final definition includes patients/persons who have lived for 2 or more years
• With incurable metastatic cancer; OR
• With incurable hematological malignancies; OR
• With locally advanced cancer that is not amenable to curative treatment (e.g., a tumor invading major blood vessels); OR
• And patients/persons living exceptionally long for their specific cancer type, even if they do not fit the primary criteria of 2 or more years after diagnosis.
Additional remarks on terminology and definition:
• No upper limit is set for the ⩾2-year survival criterion.
• Patients/persons with treatable metastases are not included in the definition (e.g., oligometastases eligible for curative treatment or metastatic testicular cancer).
• Patients/persons without metastases who cannot undergo curative treatment (e.g., due to comorbidities) or choose not to (for personal reasons) are not included in the definition, as they live with cancer but not with incurable cancer.

#### Terminology

In Round 1, healthcare professionals and stakeholders assessed five items, achieving consensus on three items, while two were deemed relevant, and patients/informal caregivers assessed four items, achieving consensus on one item and rating three as relevant (Supplemental File 2).

Based on comments, terminology items were consolidated in Round 2. Furthermore, preferences for *patients* versus *persons* were assessed. No consensus emerged on exclusive usage, allowing both terms to be used interchangeably (Table 3). After expert consultations’ review and refinement, two final terminology options remained. In Round 3, 88% of the panel (range: 76%–97%) reached consensus on the preferred terminology: *Patients living long-term with incurable cancer*. A native speaker consultation finalized the English translation.

## Discussion

In this modified hybrid Delphi study, we developed and propose a consensus-based framework for patients living long-term with incurable cancer. Through focus groups, we captured diverse perspectives on the conceptual boundaries of “long-term” and “incurable,” as well as on preferred terminology. These insights informed a structured Delphi process through which consensus was reached on key inclusion criteria: Patients living for two or more years with incurable metastatic, hematological, or locally advanced cancer, or with exceptionally long survival despite not meeting the 2-year criterion. Consensus was also achieved on the preferred terminology *patients living long-term with incurable cancer*. By involving a broad range of stakeholders, the resulting framework integrates both clinical and lived perspectives.

Several clarifications are important when applying this definition. First, no upper limit is imposed on the ⩾2-year survival criterion. However, patients with potentially curable metastases, such as those with metastatic testicular cancer, are excluded. Similarly, individuals without metastases who either cannot (e.g., due to comorbidities) or choose not to undergo curative treatment do not fall within the scope of this definition.

To our knowledge, no previous study has systematically defined this patient population using a structured, multi-round consensus approach. The Delphi method is well established for reaching agreement on definitions in healthcare, and our response rates (90% in Round 2; 88% in Round 3) align with those reported in similar studies.^24,32–34^ Although only 5 of 16 initial statements were retained in the final definition, agreement levels across subpanels were consistently high (88%–97%), aligning with established consensus benchmarks.^32,35^ Furthermore, the selected terminology was endorsed by 76%–97% of participants, exceeding endorsement rates in comparable studies,^34,35^ thus further reinforcing the robustness of our approach.

Our study responds to ongoing calls for standardized definitions in this evolving field.^4,21–23^ Prior efforts have primarily focused on distinct subpopulations within the broader group of individuals with incurable cancer. For example, Burgers et al. characterized adolescents and young adults with uncertain or poor prognoses using clinical, epidemiological, and psychosocial criteria derived from literature and database research.^36^ While informative, their approach lacked stakeholder involvement and a formal consensus process. A recent Spanish study applied a modified Delphi method to define long-term survivors with metastatic lung cancer as those with ⩾2 years progression-free survival or ⩾3 years overall survival.^24^ However, that study included only healthcare professional perspectives and focused solely on metastatic lung cancer. In contrast, our study integrated qualitative data from focus groups and continuous input from a multidisciplinary expert group to iteratively refine the consensus process for patients across all incurable cancer types.

Our framework balances medical clarity with real-world applicability, ensuring relevance in both clinical practice and research. Patients and informal caregivers favored language that reflects their lived experiences, while healthcare professionals prioritize clarity and consistency. The term *patients living long-term with incurable cancer* emerged as a shared language bridging these perspectives. Our findings show that both *patients* and *persons* are acceptable: *Patients* was preferred by healthcare professionals, *persons* by patients, informal caregivers, and other stakeholders. We recommend using *patients* in clinical and academic contexts, and *persons* when communicating with the target group to acknowledge life beyond the patient role. Given the diversity in cancer types, treatment trajectories, and prognoses,^6,37^ defining this group remains inherently complex. Kolsteren et al.^18^ highlighted the inconsistency in terminology, emphasizing the need for a universally accepted term.

### Strengths and limitations

This study has several strengths. By integrating qualitative focus group insights with a structured consensus process, we thoroughly evaluated definitions and terminology. Inclusion of a diverse range of stakeholders enhanced the validity of findings, while high response rates and consistent participation across Delphi rounds reinforced the reliability and applicability of the proposed framework in clinical and research settings. To prevent dominance by any stakeholder group, consensus required ⩾70% agreement in all subpanels and the total panel. This ensured balanced representation, though it led to more selective inclusion of items.

However, limitations remain. Patients with incurable hematological malignancies were not represented in the focus groups, potentially narrowing perspectives. Although this subgroup was represented in the expert group, direct input from these patients is needed to validate the findings. While the study was conducted in the Dutch healthcare context, its basis in a Western system supports potential international relevance. Nevertheless, the proposed framework may require adaptation to fit other healthcare systems, which we aim to validate in international settings. Although qualitative data interpretation remains inherently subjective, involvement of a multidisciplinary expert group ensured critical reflection throughout.

### Impact on healthcare

The proposed framework provides a foundation to improve recognition of, and communication with, patients living long-term with incurable cancer and to facilitate future research. In clinical practice, a shared language may support timely identification of this group, enabling more person-centered care planning and communication. By clarifying this group’s place within the broader palliative trajectory, the framework may also support more proactive and needs-based palliative care integration. It may also enable more consistent registry coding, both to document when patients meet this definition in clinical records and to facilitate identification of this population in healthcare datasets. Further studies should explore epidemiological characteristics and assess implications for patient and informal caregiver outcomes, care coordination, and healthcare policy, preferably using an (adapted) standardized framework to delineate the patient population. Longitudinal research could evaluate implementation across care settings, while international validation could determine the framework’s relevance across diverse cultural and healthcare systems. Key next steps include estimating the size of this population and examining their quality of life and care experiences. Cross-language applicability should also be explored to ensure conceptual alignment. We view the framework as a foundation that will require periodic updates over time, particularly as cancer treatments evolve and new disease trajectories emerge, to ensure it remains clinically relevant.

## Conclusion

The growing population of patients living long-term with incurable cancer occupies a unique position between survivorship and palliative care. Until now, the absence of standardized definitions has contributed to fragmented care. We present the first consensus-based framework incorporating both clinical and lived perspectives, a critical step toward improving care coordination, informing healthcare policies, and guiding future research.

## Supplemental Material

sj-docx-1-pmj-10.1177_02692163251400114 – Supplemental material for Defining patients living long-term with incurable cancer: A modified hybrid Delphi study Supplemental material, sj-docx-1-pmj-10.1177_02692163251400114 for Defining patients living long-term with incurable cancer: A modified hybrid Delphi study by Ruben Bouma, Mariken E. Stegmann, Natasja J. H. Raijmakers, Lia van Zuylen, Anna K. L. Reyners, Kristel van Asselt, Maatje D. A. van Gastel, Daan Brandenbarg and Olaf P. Geerse in Palliative Medicine
