# Supplementary material for: Defining patients living long-term with incurable cancer: A modified hybrid Delphi study
Source: Palliat Med. 2025 Dec 26;40(2):217–27. doi: 10.1177/02692163251400114 (PMC12852490; doi:10.1177/02692163251400114)
Supplement: sj-docx-1-pmj-10.1177_02692163251400114 – Supplemental material for Defining patients living long-term with incurable cancer: A modified hybrid Delphi study [file sj-docx-1-pmj-10.1177_02692163251400114.docx]

**Supplemental Material**

**Supplemental file 1.** Topic guide focus group sessions.

**Introduction and welcome**

- Introduction of moderator, observer, and participants
- Explanation of the focus group aim using patient case example
- Explanation of focus group structure

**Part 1: Terminology**

- Presentation of different terminology examples from the literature
- Question to initiate group discussion:
  - Which term would you personally use to describe people living long-term with cancer that can no longer be cured?
  - Why do you choose that term?
  - Would anyone in the group like to respond to this comment?
- Participants (patients and informal caregivers) select 2 preferred terms (post-it notes); healthcare professionals select via Mentimeter
- Discussion of selected terms and rationales
- Voting (😊) on preferred terms

*Extra discussion questions:*

- Reasons for rejecting other terms?
- Should healthcare professionals adopt the chosen term(s)?

**Part 2: Definitions**

*Subpart 1: 'Long-term'*

Participants indicate preferred timeframe post-diagnosis defining ‘long-term’

- Visual timeline exercise (patients/ informal caregivers: post-it notes; healthcare professionals: Mentimeter)
- Questions to initiate group discussion:
  - From what point after diagnosis do you consider someone to be living 'long-term' with incurable cancer?
  - What makes you choose that specific point in time?
  - Would anyone in the group like to respond to this comment?
- Group discussion to explore rationales and adjustments of initial views

*Extra discussion questions:*

- Prognostic thresholds for using the selected term?
- Who fits the concept of 'Tussenlander' (person in between)?

*Subpart 2: 'Incurable'*

- Questions per case vignette exercise and start group discussion:
  - Do you think this patient falls within the concept of ‘incurable cancer’? Why or why not?
  - What makes you feel this situation does or does not fit within this concept?
- Case vignette exercise exploring boundaries of ‘incurable’ definition (patients and informal caregivers:
  - Case A: Multiple metastases, no curative options
  - Case B: Hormone-sensitive metastatic breast cancer, prolonged stable condition
  - Case C: Single metastasis, curative-intent treatment available
  - Case D: Non-metastatic cancer, patient refuses curative treatment
  - Case E: Non-metastatic cancer, patient unable to undergo curative treatment
  - Case F: Metastatic cancer, potential for durable remission or cure
- Case vignette exercise exploring boundaries of ‘incurable’ definition (healthcare professionals):
  - Case A: Hormone-sensitive breast cancer with multiple bone metastases, favorable and prolonged response to anti-hormonal therapy.
  - Case B: Non-small cell lung carcinoma with a single liver metastasis; curative-intent treatment including primary tumor resection or radiotherapy and surgical removal or ablation of metastasis.
  - Case C: Ovarian cancer with widespread metastases; long-term stable response to targeted therapy after initial chemotherapy.
  - Case D: Metastatic melanoma with liver and brain metastases; sustained complete response after immunotherapy.
  - Case E: Metastatic colorectal cancer with high microsatellite instability (MSI-high); sustained long-term response to immunotherapy.
  - Case F: Non-metastatic cancer; patient declines available curative treatment due to quality-of-life concerns.
  - Case G: Non-metastatic cancer; patient unable to undergo curative treatment due to significant comorbidities.
- Each case discussed in group (patients and informal caregivers: verbally; healthcare professionals: via Mentimeter then verbal discussion)

*Extra discussion questions:*

- Do these cases exemplify a 'Tussenlander'?

**Part 3: Identifying subgroups**

- Questions for open brainstorm on criteria defining patient subgroups with distinct care needs;
  - Do you think that all people with incurable cancer have similar care needs, or are there certain groups who might require a different approach?
  - What characteristics do you think are important to distinguish between people within this group?
  - Can you give an example of a situation where someone with incurable cancer needed different support compared to someone else with the same diagnosis?
- Facilitated discussion using suggested criteria. Q: Why do you think this subgroup needs different support?
  - Active treatment vs. watchful waiting
  - Differences in life stages (young families vs. elderly, retired)
  - Cancer type
  - Treatment type (local vs. systemic)
  - Performance status (0-5 scale)
  - Treatment goal: lifespan vs. quality of life
  - Symptom burden (high vs. low)
  - Impact on daily life (high vs. low)
  - Potential distinction based on duration of illness (e.g., long-term gradients)

**Part 4: Closing**

- Final opportunity for additional comments or missed topics
- Question and answer session
- Explanation of next steps and feedback mechanism
- Thanks and conclusion

**Supplemental file 2.** Delphi items and results Round 1.

| **Round 1** | **Total panel**  **(*n*=73)** | **Patients & informal caregivers**  **(*n*=17)** | **Healthcare professionals**  **(*n*=36)** | **Relevant stakeholders**  **(*n*=20)** |
| --- | --- | --- | --- | --- |
| **Definition: Incurable** | | | | |
| Patients without metastases but with a cancer type that is locally advanced to the extent that it cannot be treated with curative intent. | 4 | 4 | 4 | 4 |
| Patients without metastases who cannot undergo curative treatment (e.g., due to comorbidities). | 4 | 4 | 4 | 3 |
| Patients without metastases who do not wish to undergo curative treatment (e.g., they prioritize quality of life). | 4 | 3 | 4 | 3 |
| Patients with oligo-metastatic disease and a good prognosis, with the possibility of undergoing a treatment with curative intent (up to a maximum of 5 metastases in addition to the primary tumor). | 2 | 2.5 | 2 | 2.5 |
| Patients with a hematologic malignancy that is no longer curable. | 4 | 4 | 4 | 4 |
| **Definition: Long-term** | | | | |
| 1. An absolute timeframe must be included in the definition. | 2 | 2 | 2 | 2 |
| 1a. Patients who are alive 1 year after the initial diagnosis. | 3 | 2 | 3 | 3 |
| 1b. Patients who are alive 2 years after the initial diagnosis. | 4 | 2.5 | 3.5 | 4 |
| 1c. Patients who are alive 5 years after the initial diagnosis. | 4 | 2.5 | 4 | 4 |
| 2. A relative timeframe per cancer type must be included in the definition. | 3 | 3 | 3 | 3 |
| 2a. Patients with a specific type of cancer who live longer than the median survival (the point at which 50% of patients are still alive). | 3 | 4 | 3 | 4 |
| 2b. Patients with a specific type of cancer who live longer than the third quartile (the point at which 25% of patients are still alive). | 3 | 4 | 3 | 4 |
| 2c. Patients with a specific type of cancer who live longer than the tenth decile (the point at which 10% of patients are still alive). | 3 | 3 | 3 | 4 |
| The definition should exclude patients with incurable cancer who show a prolonged, good response to therapy, making them potentially considered "cured" in the future. | 2 | 2 | 2 | 2 |
|  | **Total panel**  **(*n*=78)** | **Patients & informal caregivers**  **(*n*=22*)** | **Healthcare professionals**  **(*n*=36)** | **Relevant stakeholders**  **(*n*=20)** |
| **Terminology** |  |  |  |  |
| There should be two separate terms: one medical terminology and one laymen terminology. | 3 | 2 | 3 | 3 |
| **Medical terminology** |  |  |  |  |
| It is important that the term is in English, making it internationally applicable. | 3 |  | 3 | 3 |
| It is important that the term is in Dutch but easily translatable into English, making it internationally applicable. | 4 |  | 4 | 4 |
| The term must contain at least one word related to the concept of prolonged/long-lived. | 4 |  | 4 | 4 |
| The term must contain at least one word related to the concept of advanced/incurable | 4 |  | 4 | 4 |
| **Laymen terminology** |  |  |  |  |
| It is important that the term is in English, making it internationally applicable. |  | 2 |  |  |
| It is important that the term is in Dutch, making it easy to use in daily conversation. |  | 4 |  |  |
| It is important that the term does not contain words associated with stigma (such as “cancer” or “palliative”). |  | 3 |  |  |

* Responses from healthcare professionals (n = 2) and stakeholders (n = 3) who were also informal caregivers are included in both the laymen and medical terminology categories.

**Supplemental file 3.** Delphi items and results Round 3.

| **Round 3** | **Total panel**  **(*n* = 64)** | **Patients & informal caregivers**  **(*n* = 15)** | **Healthcare professionals**  **(*n* = 32)** | **Relevant stakeholders**  **(*n* = 17)** |
| --- | --- | --- | --- | --- |
| **Terminology**  *Do you agree with the use of one or more of the following terms?*  *Multiple options may be considered appropriate* | | | | |
| Patients with long-term incurable cancer | 28% | 20% | 31% | 29% |
| Patients living long-term with incurable cancer | 88% | 80% | 97% | 76% |
| I do not find either term appropriate | 5% | 7% | 0% | 12% |
| **Definition**  *Do you agree with the proposed definition?* | | | | |
| Yes | 94% | 93% | 97% | 88% |
| No | 6% | 7% | 3% | 12% |
